# O22 - Asian Americans demonstrate optimal compliance in CDC recommended pediatric vaccine schedule: implication of immunisation in autism causal inference

**DOI:** 10.1186/2045-7022-4-S1-O22

**Published:** 2014-03-14

**Authors:** Larry Holmes, Patricia Oceanic, Diane Fitzgerald, Kelli Grant, Alexandra Lahurd, Kirk Dabney

**Affiliations:** 1 Office of Health Equity and Inclusion, Nemours / A.I.duPont Children Hospital, Wilmington, DE, USA

## Purpose

The Center for Disease Control and Prevention (CDC) provides the guidelines and recommendations for age-specific immunization schedule. We aimed to assess the prevalence of vaccination schedule by race/ethnicity, and to determine whether or not Asians demonstrate optimal compliance.

## Methods

The CDC recommends one dose of diphtheria toxoids, acellular pertusis and tetanus (Tdap), and pneumococcal vaccines (PCV), two doses of varicella, measles, mumps and rubella (MMR) and three doses of Hepatitis B (HBV), and inactivated poliovirus to be received by age 11-12 years. We assessed age-specific recommendation adherence. Data were examined cross-sectionally on vaccination received during 2010. Chi squared statistic and multivariable logistic regression model were used.

## Results

Recipients were Whites/Caucasians, 1,917 (32.7%), Blacks/African Americans (AA), 2904 (49.5%), Asian, 134 (2.3%), Hawaiian native /Pacific Islander,4 (0.1%), American Indian/Alaskan Native (AI/AN),9(0.2%), and some other race, 727(12.4%). There was overall 92.3% compliance to the recommended schedule. A significant racial variability in MMR as well as HBV were observed, Asian (98.5%), AA (98.4%) and Caucasian (97.1%), χ^2^(7)=20.6, *p*=0.01, and Asians (99.3%), AA(94%) and Caucasian (98.7%), χ^2^(7)=23.9, *p*=0.001 respectively. Asians demonstrated highest compliance in the receipt of varicella (Asians [99.3%], AA [98.6%], and Caucasian [97.1%], χ^2^(7)=18.7, *p*=0.01, and toxoid poliovirus (Asians [100%], AA [99.4%] and Caucasian [99%], χ^2^(7)=12.3, *p*=0.09. Asians (97.0%) relative to AA (93.1%) and Caucasian (91%) demonstrated the highest compliance in all vaccines combined, χ^2^(7)=24.5, *p*=0.001. Caucasians and AA, relative to Asians were 69% (Odds ratio [OR]=0.42, 95% CI, 0.15-1.14, and 58%, (OR=0.31, 95%CI, 0.11-0.85) less likely to adhere to the CDC schedule respectively. However, multivariable model indicated insignificant racial disparities between Asians and Caucasians, adjusted OR, 0.45, 99%CI, 0.08-1.11.

## Conclusion

Asians demonstrated highest compliance, indicative of racial/ethnic disparities in immunization schedule as well as ecologic lack of the causal inference on the role of immunization in autism, given the lowest prevalence of autism among Asian Americans.

